# Lipid rafts are essential for release of phosphatidylserine-exposing extracellular vesicles from platelets

**DOI:** 10.1038/s41598-018-28363-4

**Published:** 2018-07-03

**Authors:** Hao Wei, Jean-Daniel M. Malcor, Matthew T. Harper

**Affiliations:** 1 Department of Pharmacology, University of Cambridge, Cambridge, United Kingdom; 2 Department of Biochemistry, University of Cambridge, Cambridge, United Kingdom

## Abstract

Platelets protect the vascular system during damage or inflammation, but platelet activation can result in pathological thrombosis. Activated platelets release a variety of extracellular vesicles (EVs). EVs shed from the plasma membrane often expose phosphatidylserine (PS). These EVs are pro-thrombotic and increased in number in many cardiovascular and metabolic diseases. The mechanisms by which PS-exposing EVs are shed from activated platelets are not well characterised. Cholesterol-rich lipid rafts provide a platform for coordinating signalling through receptors and Ca^2+^ channels in platelets. We show that cholesterol depletion with methyl-β-cyclodextrin or sequestration with filipin prevented the Ca^2+^-triggered release of PS-exposing EVs. Although calpain activity was required for release of PS-exposing, calpain-dependent cleavage of talin was not affected by cholesterol depletion. P2Y_12_ and TPα, receptors for ADP and thromboxane A_2_, respectively, have been reported to be in platelet lipid rafts. However, the P2Y_12_ antagonist, AR-C69931MX, or the cyclooxygenase inhibitor, aspirin, had no effect on A23187-induced release of PS-exposing EVs. Together, these data show that lipid rafts are required for release of PS-exposing EVs from platelets.

## Introduction

Platelets protect the vascular system during damage or inflammation, but inappropriate or excessive platelet activation results in pathological thrombosis^1^. Platelet activation and thrombosis on a ruptured atherosclerotic plaque in a coronary artery is the major precipitating event in acute coronary syndromes (ACS), such as unstable angina and myocardial infarction. Anti-platelet drugs are therefore used during ACS to prevent of further thrombotic events^2^. These anti-platelet drugs include the cyclooxygenase inhibitor, aspirin, and P2Y_12_ antagonists, such as clopidorgel, prasugrel, ticagrelor or cangrelor^2–4^.

Activated platelets release a variety of extracellular vesicle (EVs). These have been variously categorised based on their size, surface markers, and mechanism of release. EVs that are shed from the plasma membrane have been variously called ‘microparticles’, ‘shedding microvesicles’ or ‘ectosomes’^5^. EVs shed from the plasma membrane often expose phosphatidylserine (PS) on their outer leaflet. In non-activated platelets, PS is asymmetrically restricted to the inner leaflet. An increase in intracellular Ca^2+^ concentration ([Ca^2+^]_i_) during platelet activation can trigger loss of plasma membrane asymmetry and exposure of PS in the outer leaflet^6^. PS exposure may contribute to shedding of EVs^7^. In contrast, vesicles formed by inward budding of intracellular granule membranes, and released by secretory granule exocytosis, have been termed ‘exosomes’^8^. Although exosomes are generally considered to not expose PS, a recent study suggests that exosomes might also expose PS^9^. However, since there has not been consistent use of these terms, we use the more neutral term, ‘extracellular vesicle’ (EV)^5^.

PS-exposing EVs are pro-thrombotic. PS forms a pro-coagulant binding site for the tenase and prothrombinase coagulation complexes. PS-exposing EVs increase the rate and extent of thrombin generation, promoting thrombosis^10^. They also regulate wound healing, inflammation and vascular integrity^11,12^. Circulating PS-exposing EVs are elevated in many cardiovascular and metabolic disorders, including atherosclerosis, ACS, hypertension, heart failure, type II diabetes, and obesity^13–22^. In addition, platelet-derived EVs have been linked to tumour progression and metastasis^23,24^. These associations make PS-exposing EVs both attractive therapeutic targets and potential biomarkers of disease progression^25^.

The mechanisms by which PS-exposing EVs are shed from activated platelets are not well characterised. PS exposure and EV shedding can be triggered by an increase in [Ca^2+^]_i_^26^. EV shedding is inhibited in patients with Scott syndrome, who are deficient in ability to expose PS, indicating that PS exposure is an important part of the molecular mechanism^27^. In addition, cytoskeletal disruption by the Ca^2+^-dependent protease, calpain, is required^7,28,29^. Beyond these events, little is known about how the process of shedding PS-exposing EVs from platelets is regulated.

Cholesterol-rich lipid rafts provide a platform for coordinating signalling in platelets and other cells^30^. Lipid rafts are involved in PS-exposing EV release from monocytes^31^, endothelial cells^32^ and erythrocytes^33,34^. In this study, we investigated whether lipid rafts are required for platelets to release PS-exposing EVs.

## Results

### The Ca^2+^ ionophore, A23187, triggers release of PS-positive platelet-derived EVs

**Figure 1:**
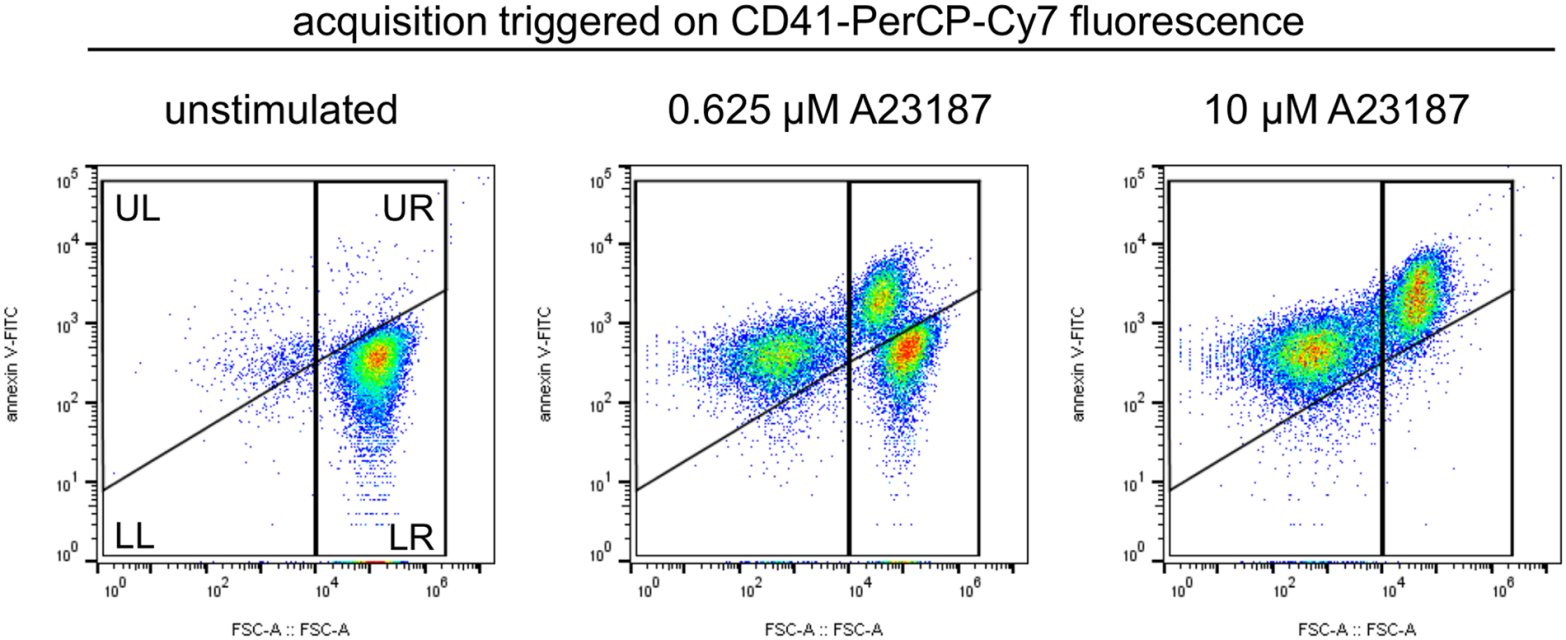
The Ca^2+^ ionophore, A23187, triggers release of PS-exposing EVs from platelets. Washed platelets were stimulated with the indicated concentration of A23187 for 10 minutes, after which samples were stained with anti-CD41-PerCP-Cy7, and annexin V-FITC to detect PS exposure. PerCP-Cy7 fluorescence was used to trigger acquisition of CD41^+^ events. The panels show density plots of events from low density (blue) to high density (red) of forward scatter (FSC-A) and FITC fluorescence. Unstimulated platelets have high FSC-A and low annexin V-FITC binding (LR). Stimulation with A23187 triggered PS exposure in platelets (UR) and release of PS-exposing EVs (UL). The vertical line separating left and right was defined by the FSC-A of 1 µm silica beads. The density plots are representative of data from 6 different donors.

Washed platelets were stimulated with the Ca^2+^ ionophore, A23187, to trigger release PS-exposing EVs. A Ca^2+^ ionophore was used rather than physiological agonists in order to bypass platelet receptors and Ca^2+^ entry channels, which may be affected by cholesterol depletion. Platelet-derived PS-exposing EVs were defined as CD41^+^/annexinV^+^ events that were smaller than 1 µm (Fig. 1). We acknowledge that the number of events seen by this approach is likely to be an underestimate of the total number of platelet-derived PS-exposing EVs as we will predominantly detect the largest microparticles. Platelets exposed PS and released PS-exposing EVs in response to increasing concentrations of A23187 (Fig. 1).

### Depletion of platelet cholesterol prevents release of PS-positive EVs

**Figure 2:**
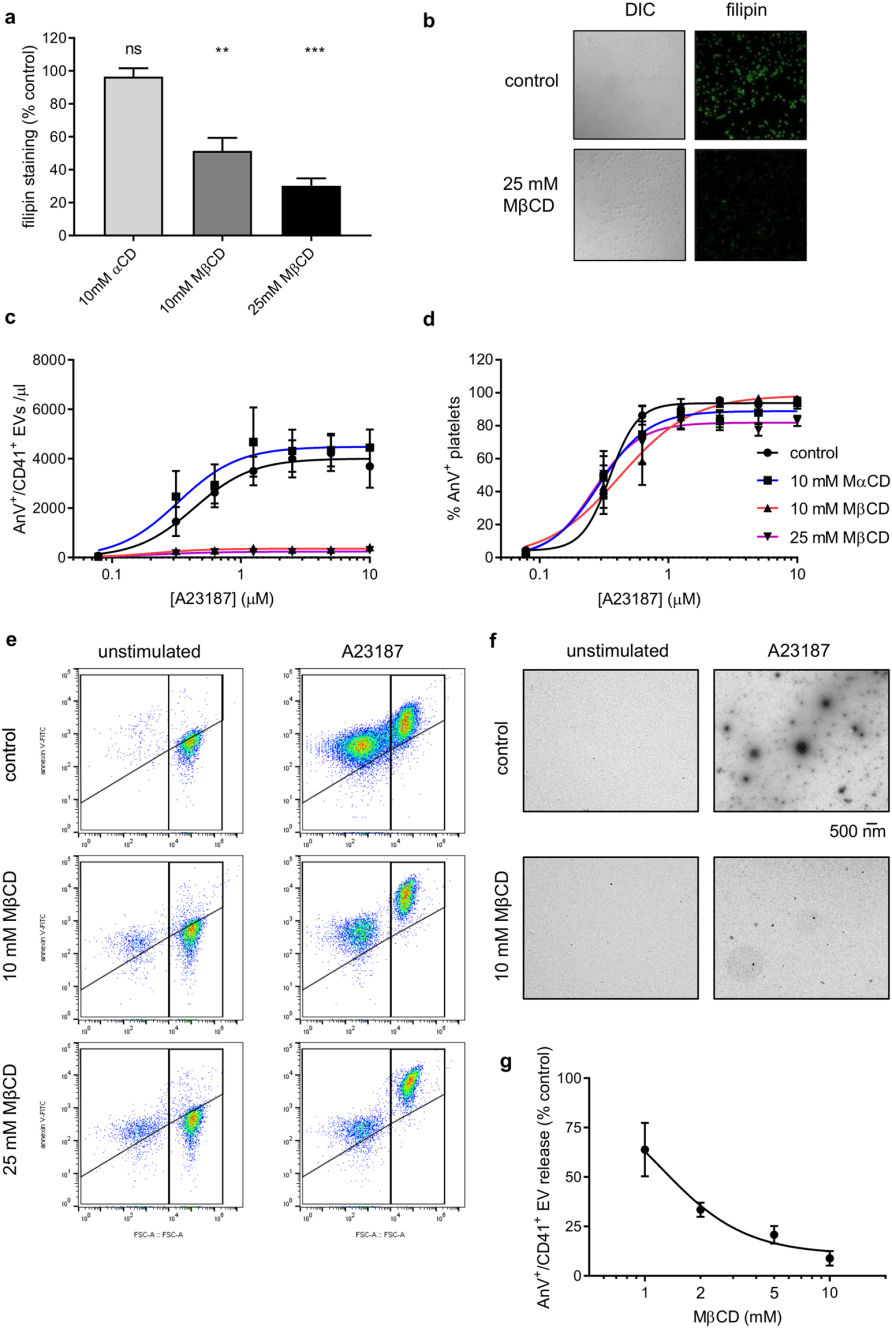
Cholesterol depletion inhibits release of PS-exposing EVs. (**a**) Platelets were treated with MβCD (10 mM or 25 mM), αCD (10 mM) or vehicle as control, then fixed and stained with filipin. Filipin fluorescence was determined by confocal microscopy. (n = 5; *p < 0.05; **p < 0.01; ns, not significantly different from control). (**b**) Representative images of filipin-stained platelets. (**c**) Following treatment with MβCD, MαCD or vehicle, platelets were stimulated with various concentrations of A23187 for 10 minutes. EVs were measured as in Fig. 1. (**d**) The percentage of platelets that stained positive for annexin V. (Mean ± SEM; n = 5). (**e**) The effect of different concentrations of MβCD on release of PS-exposing EVs in response to 10 µM A23187 (Mean ± SEM; n = 5). (**f**) Representative density plots for platelets stimulated with 10 µM A23187.

To test whether lipid rafts are required for PS-positive EV release, cholesterol was depleted from washed platelets by treatment with methyl-β-cyclodextrin (MβCD). Following treatment with MBCD, platelets were fixed with PFA then stained with filipin, which binds to cholesterol with high affinity^35^. Filipin fluorescence decreased following treatment with MβCD in a dose-dependent manner (Fig. 2a,b). In some previous studies, MβCD was incubated in platelet-rich plasma (PRP) to deplete cholesterol prior to isolation of washed platelets (e.g.^36^). However, in preliminary studies we found no additional benefit of this approach in terms of cholesterol depletion, but also observed that it reduces platelet recovery after centrifugation from PRP, which may suggest an increase in mechanical fragility of the platelets. α-cyclodextrin (αCD), which does not remove cholesterol from membranes^30^, did not decrease filipin fluorescence, indicating the specificity of MβCD (Fig. 2a,b).

When platelets treated with MβCD (10 or 25 mM) were stimulated with A23187, no PS-exposing platelet-derived EVs were detected (Fig. 2c). In contrast, PS exposure in intact platelets (i.e. >1 µm) was unaffected, suggesting that platelet activation and the exposure of negatively-charged phospholipids was not affected (Fig. 2d). Consistent with this, we did not observe an increase in annexinV negative EVs (CD41^+^, <1 µm) in MβCD-treated samples following A23187 stimulation (see representative density plots in Fig. 2e), indicating that the lack of PS-exposing EVs in these samples was not a result of inhibited PS exposure in the EVs themselves (and hence not detected as AnV^+^/CD41^+^ events). α-cyclodextrin (10 mM) had no effect on release of PS-exposing EVs or on platelet PS exposure (Fig. 2c,d). α -cyclodextrin has a similar structure to MβCD but has a much lower affinity for cholesterol. It can therefore be used as a control for any cholesterol-independent effects of MβCD. Representative density plots are shown in Fig. 2e. To confirm the flow cytometry data, the EVs were isolated by centrifugation and visualised by transmission electron microscopy (Fig. 2f). The inhibitory effect of MβCD was concentration-dependent, with less effect at 1–5 mM (Fig. 2g).

The effect of MβCD on plasma membrane integrity was assessed using a fluorescent fixable viability dye (FVD). This reagent is cell-impermeable so can be used to label cells that have lost plasma membrane integrity. Heat-killed platelets showed strong FVD fluorescence (Supplementary Fig. 1). In contrast, neither MβCD treatment, nor stimulation with A23187 for 10 minutes, increased FVD fluorescence in platelets. The effect of MβCD on release of PS-exposing EVs is not due to platelet death. Consistent with this, re-plotting of the data in Fig. 2c,d to give PS-exposing EVs per platelet, or PS-exposing EVs per PS-exposing platelet (Supplementary Fig. 2), shows that the effect of MβCD on EV release is not due to reduced platelet viability or reduced platelet activation.

**Figure 3:**
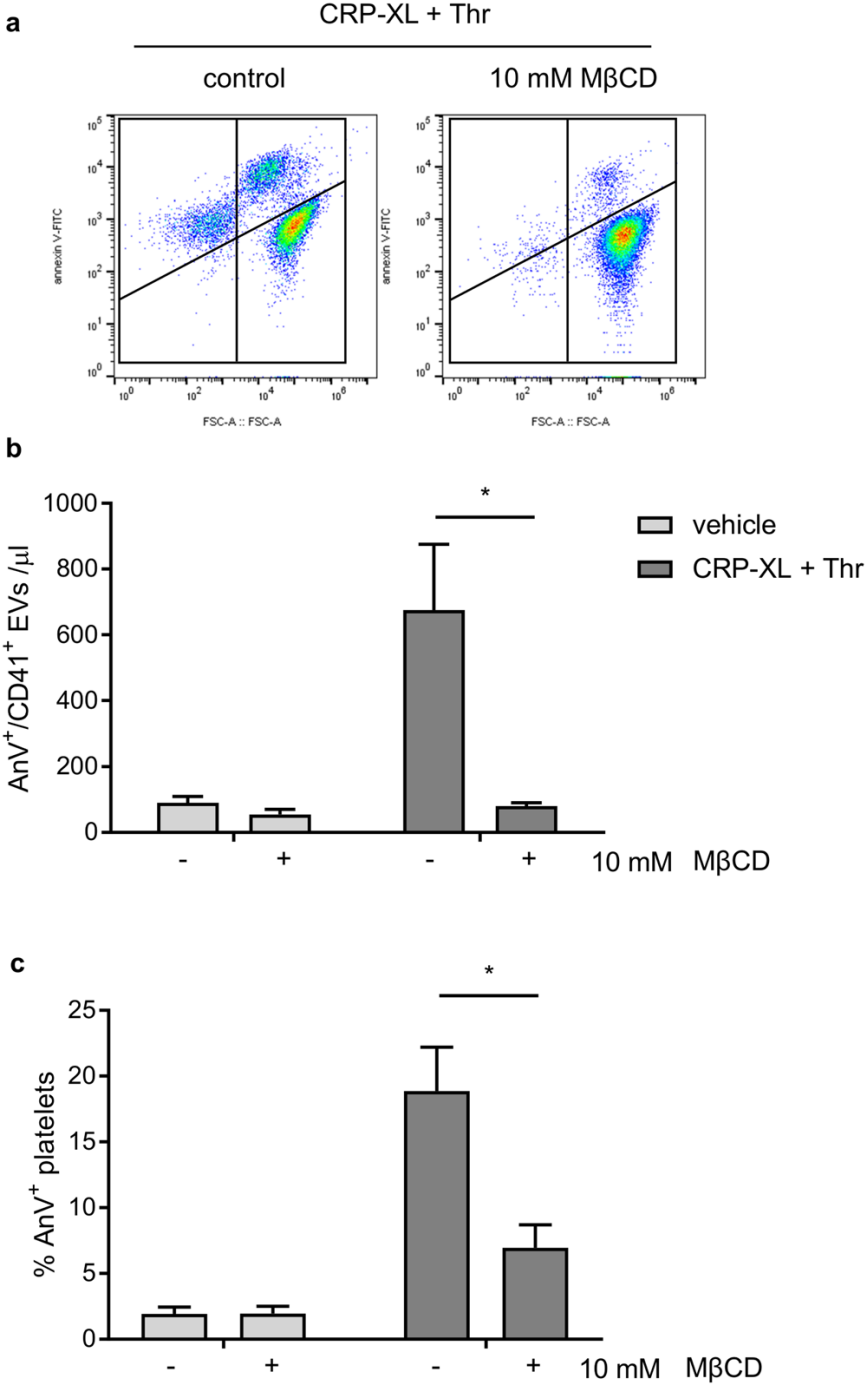
Cholesterol depletion inhibits release of PS-exposing EVs in response to physiological stimulation. Platelets were treated with MβCD (10 mM) or vehicle as control, then stimulated with CRP-XL (10 µg/ml) plus thrombin (1 U/ml). (**a**) EVs were measured as in Fig. 1. (**b**) The percentage of platelets that stained positive for annexin V. (Mean ± SEM; n = 5). (**c,d**) Representative density plots from these experiments.

Although A23187 effectively stimulated EV release from platelets, it is possible that the mechanism of EV release in response to A23187 does not mimic the mechanisms by which EVs are released in response to physiological platelet activators. To test this, platelets were stimulated with a mix of cross-linked collagen-related peptide (CRP-XL) and thrombin. This dual stimulation promotes PS exposure and release of PS-exposing EVs. Pre-treatment with MβCD (10 mM) inhibited release of PS-exposing EVs (Fig. 3a,b), but also inhibited PS exposure in platelets (Fig. 3c), suggesting that platelet receptors or Ca^2+^ channels may also be affected by cholesterol depletion (see Discussion). For this reason, we continued with A23187 rather than more physiological activators.

**Figure 4:**
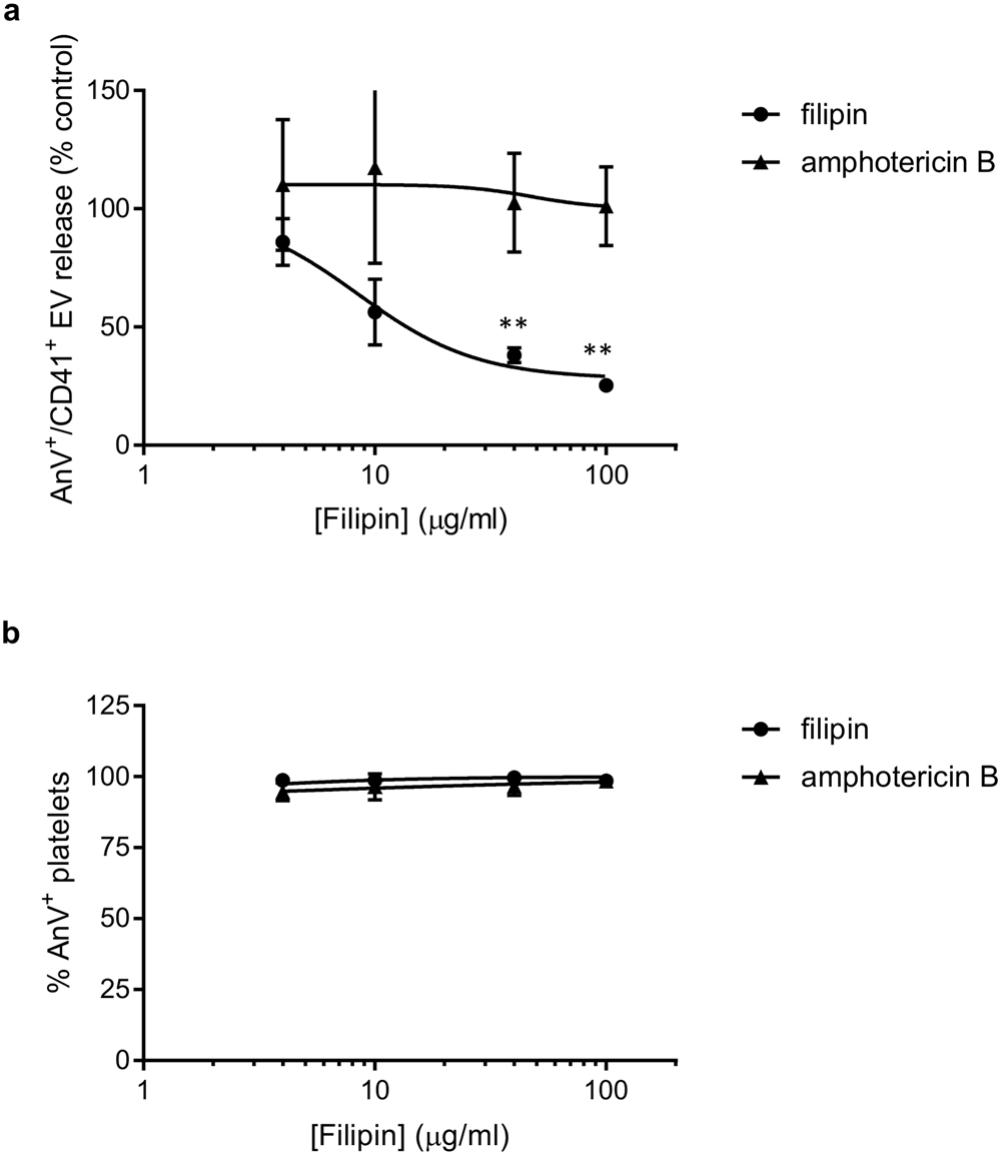
Filipin, but not amphotericin B, inhibits release of PS-exposing EVs. (**a,b**) Washed platelets were treated with the indicated concentrations of filipin or amphotericin B, or vehicle as control prior to stimulation with A23187 (10 µM). CD41^+^/annexin V^+^ EVs were identified as in Fig. 1. (**a**). EVs release in (**a**) are expressed as % of vehicle-treated, A23187-stimulated platelets (n = 5; **p < 0.01). (**b**) Shows the percentage of annexin V-positive platelets from these experiments.

Filipin, when applied to unfixed cells, can sequester cholesterol and disrupt lipid rafts. Platelets treated with filipin before stimulation with A23187 released significantly fewer detectable PS-exposing EVs (Fig. 4a; n = 5; **p < 0.01), without effect on PS exposure (Fig. 4b). In contrast, amphotericin B, a structurally-related polyene macrolide antibiotic, had no effect on release of PS-exposing EVs over the same range of concentrations. Amphotericin B is selective for ergosterol over cholesterol^37^. Together, these data suggest that disruption of membrane cholesterol prevents the release of PS-exposing EVs from platelets.

### Cholera toxin B, a marker of lipid rafts, binds to platelet-derived EVs

**Figure 5:**
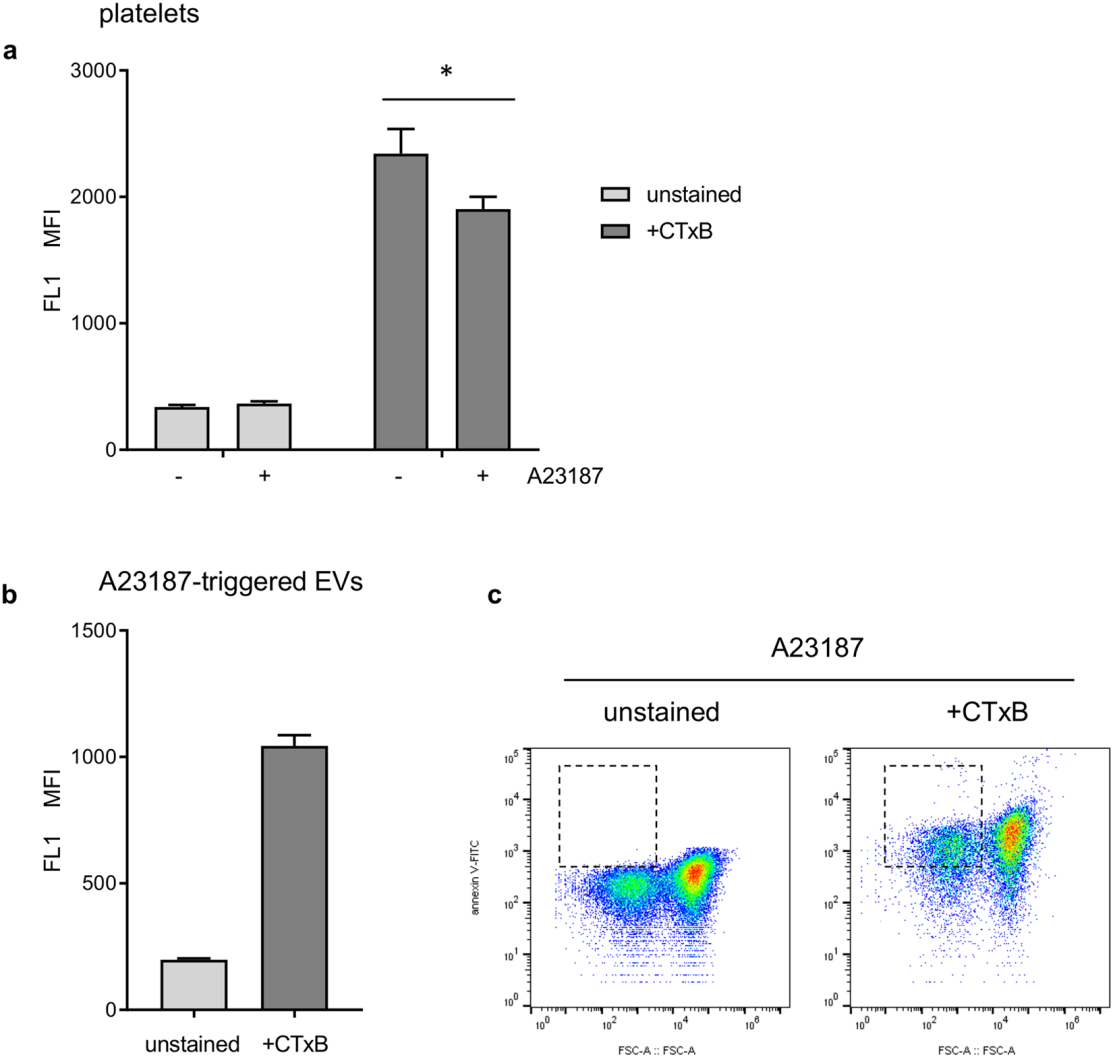
Cholera toxin B (CTxB) binds to platelets and platelet-derived EVs. (**a**) Platelets, unstimulated (−) or treated with A23187 (+) were stained with FITC-conjugated CTxB then measured by flow cytometry. Median fluorescence intensity (MFI) in FL1 is shown for stained and unstained platelets. A23187 stimulation leads to a significant reduction in CTxB binding (n = 5; *p < 0.05). (**b**) MFI in FL1 for EVs in the same experiments.

Cholera toxin B (CTxB) binds to GM1 ganglioside, a widely-used marker of lipid rafts^38^. FITC-conjugated CTxB binds to unstimulated platelets (Fig. 5a). Following stimulation with A23187, the median fluorescence intensity of CTxB-FITC bound to intact platelets decreased (specific staining reduced to 78.4 ± 6.0% of matched controls; p < 0.05, n = 5; Fig. 5a). In addition, most platelet-derived EVs stained positive for CTxB-FITC (Fig. 5b). In this experiment, CTxB-FITC fluorescence was recorded through FL1, so annexin V-FITC was omitted. However, as shown in Fig. 1, almost all platelet-derived (CD41^+^) EVs that we could detect were annexin V positive. 74.7 ± 0.8% (n = 5) of CD41^+^ EVs detected stained positive for CTxB-FITC (indicated by gate in Fig. 5c). This suggests that GM1 is released from platelets in EVs and supports the hypothesis that EVs are released from sites of lipid rafts.

### Cholesterol depletion does not affect calpain activity

**Figure 6:**
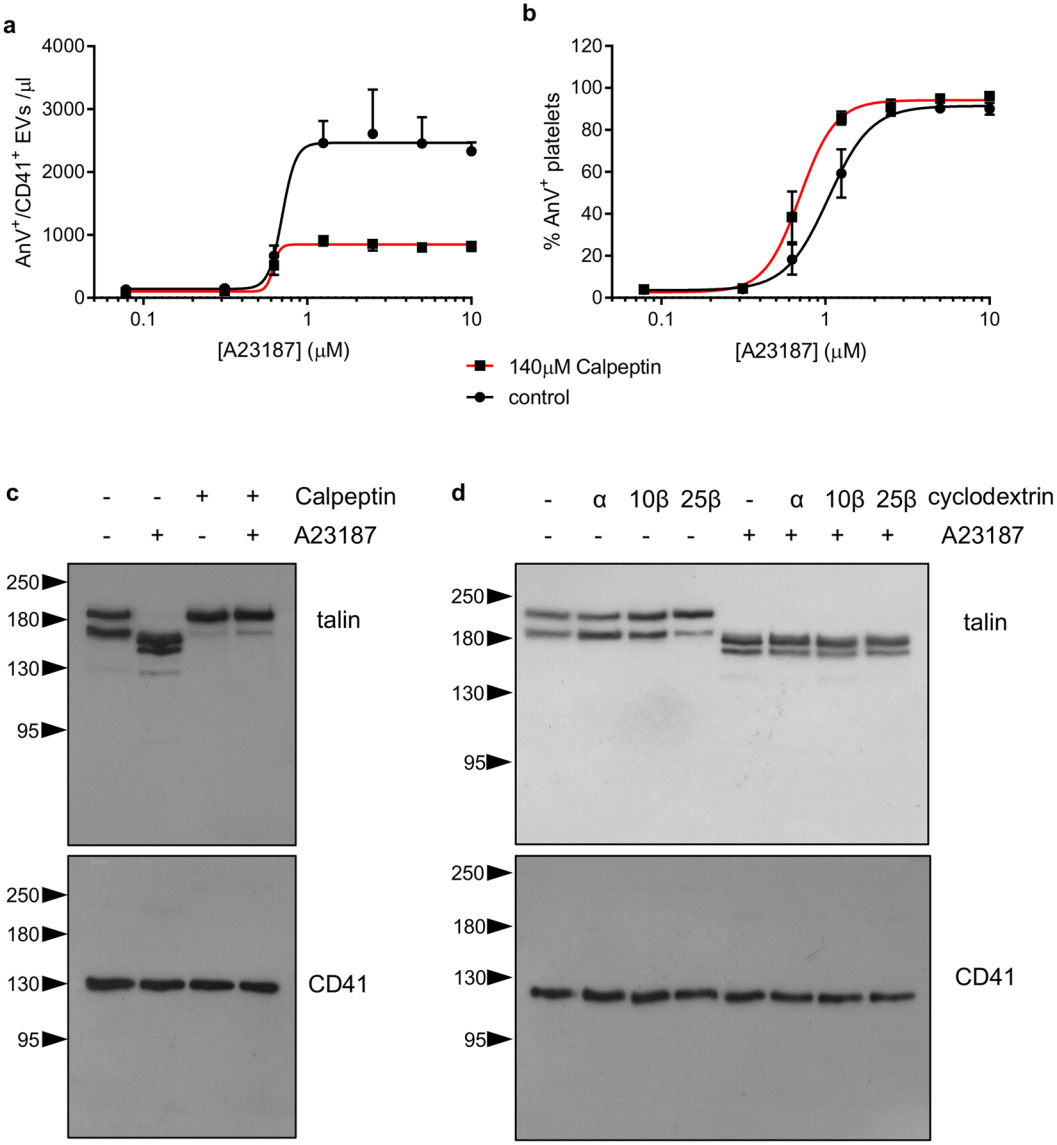
Cholesterol depletion does not affect calpain activity. (**a,b**) Platelets were treated with the calpain inhibitor, calpeptin (140 µM) or the vehicle (DMSO) prior to stimulation with A23187. Number of PS-exposing EVs in (**a**) and percentage of platelet exposing PS is shown in (**b**) (n = 5). (**c**) Platelets were treated with calpeptin (or vehicle) then A23187, as indicate, then lysed. Proteins were separated by SDS-PAGE and talin detected with a specific antibody. The membrane was then stripped and re-probed with an anti-CD41-antibody. A23187-triggered talin cleavage was inhibited by calpeptin. The blots are representative of 5 independent experiments. (**d**) Platelets were treated as indicated with 10 mM MβCD (10β) 25 mM MβCD (25β), 10 mM αCD (10α), or vehicle as control (−), prior to stimulation where indicated. A23187-triggered talin cleavage was not affected by MβCD.

Calpain, a Ca^2+^-dependent protease, is important for release of PS-exposing EVs from platelets^28,29^. We confirmed this in our experimental system by treating platelets with calpeptin, a calpain inhibitor (140 μM), which significantly reduced the release of PS-exposing EVs (Fig. 6a). Calpeptin increased the proportion of platelets that bound annexin V at lower concentrations of A23187 (Fig. 6b), consistent with a previous report^39^. Stimulation with A23187 led to cleavage of talin, which was prevented by prior treatment with calpeptin (Fig. 6c), confirming that talin is a calpain substrate in platelets. In contrast, cholesterol depletion by MβCD had no effect on talin cleavage (Fig. 6d). This suggests that cholesterol depletion does not affect Ca^2+^-dependent activation of calpain in platelets.

### P2Y_12_ and thromboxane signalling are not required for A23187-triggered release of PS-exposing EVs

**Figure 7:**
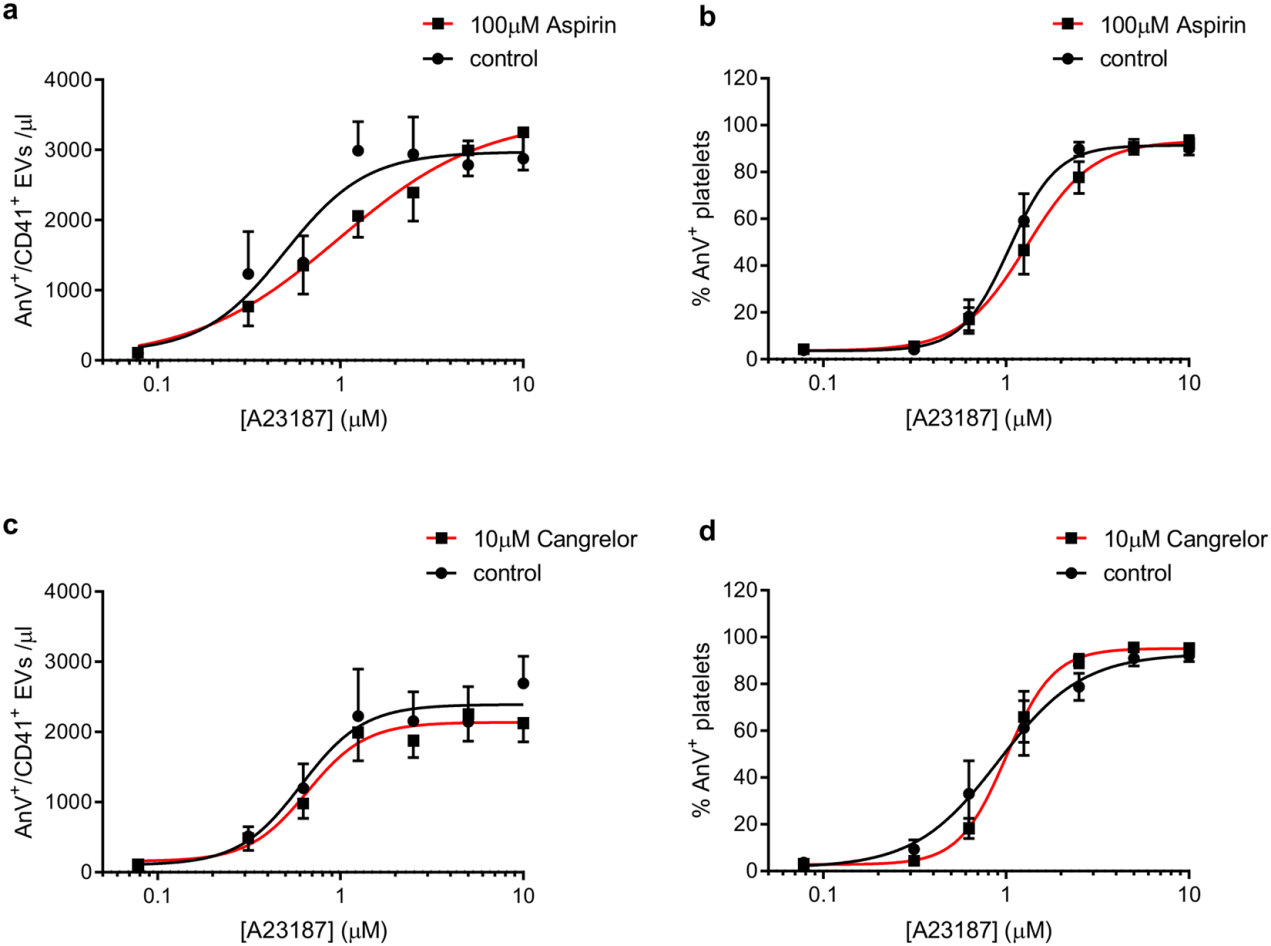
P2Y_12_ and thromboxane signalling are not required for release of PS-exposing EVs. Platelets were treated with the P2Y_12_ antagonist, cangrelor (AR-C 69931MX, 10 µM; **a**,**b**) or the cyclooxygenase inhibitor, aspirin (100 µM; **c**,**d**) prior to stimulation with A23187. Release of PS-exposing EVs is shown in (**a,c**); PS exposure in platelets is shown in (**b,d**). Data are mean ± SEM (n = 5).

The ADP receptor, P2Y_12_, and the thromboxane receptor, TPα, have been reported to reside in lipid rafts^36,40,41^. To determine whether disruption of either of these pathways could account for the effect of cholesterol depletion, platelets were treated with the P2Y_12_ antagonist, AR-C 69931MX (Cangrelor, 10 µM) or the cyclooxygenase inhibitor, aspirin (100 µM). Neither drug had any significant effect on platelet PS exposure or release of PS-exposing EVs (Fig. 7a–d).

## Discussion

Platelet-derived PS-exposing EVs are pro-thrombotic, pro-inflammatory, and associated with cardiovascular and metabolic disease^11^. In this study we show that intact cholesterol-rich lipid rafts are required for platelets to release PS-exposing EVs. MβCD, which is widely used to disrupt lipid rafts, depleted cholesterol from platelets and prevented the release of PS-exposing EVs from platelets. Filipin, which binds and sequesters membrane cholesterol, also prevented release of PS-exposing EVs. In contrast, the related polyene antibiotic, amphotericin B, had no effect. Cholera toxin B (CTxB) binds to the lipid raft marker, GM1 ganglioside. CTxB readily bound to platelet EVs, whereas CTxB binding of platelets was reduced by treatment with A23187, suggesting that GM1 ganglioside is lost from platelets during EV shedding. Together, these data indicate that lipid rafts have an essential role in the release of PS-exposing EVs from platelets.

The role of lipid rafts in formation and release of PS-exposing EVs is consistent with many other cell types. Erythrocytes treated with Ca^2+^ ionophores release PS-exposing EVs that are enriched in the lipid raft marker, stomatin^33,34^. Lipid rafts are required for monocytes to release tissue factor (TF)-rich EVs^31^. Lipid rafts also control the protein composition of these monocyte-derived EVs, by allowing incorporation of TF, PGSL-1 and β1 integrins, but excluding CD45^31,42^. The role of lipid rafts is also consistent with the report that platelet-derived EVs are enriched in cholesterol relative to unstimulated platelets^43^.

The molecular mechanisms that control PS-exposing EV release in platelets are poorly understood, especially when considered in comparison to our detailed knowledge of other platelet effector processes, such as integrin α_IIb_β_3_ activation or granule secretion^44–47^. A rise in [Ca^2+^]_i_ leads to calpain activation and cleavage of cytoskeletal proteins, such as talin^7,28,29^. This may uncouple the plasma membrane from the cytoskeleton. In addition, the increased [Ca^2+^]_i_ triggers exposure of PS in the outer leaflet of the plasma membrane through activation of the phospholipid scramblase, TMEM16F^7^. PS exposure appears to be important for EV release since PS-exposing EVs release is reduced in Scott Syndrome^27^, a rare bleeding disorder caused by mutations in TMEM16F^48,49^. Similarly, PS-exposing EVs release was reduced in *Tmem16f*^−/*−*^ platelets^50^. PS exposure may promote EV release by altering membrane curvature^51^. However, beyond calpain and PS exposure, little is known about how platelets generate and release PS-exposing EVs.

How lipid rafts contribute to release of PS-exposing EVs is still unclear. Lipid raft disruption does not appear to disrupt PS exposure, since MβCD or filipin had no effect on annexin V binding to stimulated platelets. The loss of PS-exposing EVs is also not due to a selective deficit of PS exposure in EVs, since we do not see an increase in annexinV negative EVs following stimulation of MβCD-treated samples. In addition, lipid raft disruption does not appear to affect calpain activity, since calpain-dependent cleavage of talin was unaffected. Although the receptors P2Y_12_ and TPα have been found in platelet lipid rafts, neither inhibition of P2Y_12_ nor inhibition of thromboxane synthesis with aspirin affected PS exposure or release of PS-exposing EVs. In contrast, previous reports have indicated a role for P2Y_12_ in the release of PS-exposing EVs when platelets are stimulated through cell surface receptors^52,53^. This implies that P2Y_12_ enhances signalling downstream of receptor stimulation, perhaps by promoting intracellular Ca^2+^ signalling, rather than being involved in the EV release mechanism itself.

Lipid rafts may serve to localise the required molecular machinery for PS-exposing EV release but, beyond calpain and TMEM16F, this machinery is not known. One reason for this sparse information may be the relative difficulty in studying EVs. In this study, we have mostly used flow cytometry to demonstrate the presence of PS-exposing EVs. This is not without pitfalls. Extracellular vesicles (EVs) are believed to be heterogeneous population from 100 nm to 1 µm. Flow cytometry can struggle to resolve the smaller EVs^5,54^, and our measurement of PS-exposing EVs is likely to be an underestimate because we will predominantly be detecting the largest EVs. Platelet-derived EVs are also heterogeneous in their mechanism of formation, that is, whether they are derived from outward blebbing or budding from the plasma membrane (sometimes called microparticles, or ectosomes), or are formed by endocytosis into secretory granules and are released by subsequent exocytosis (sometimes called exosomes)^5^. The microparticles/ectosomes are thought to be the larger particles and more likely to expose PS, whereas the exosomes are thought to be smaller and not to expose PS^8^. In this study we are focusing on the larger, PS-exposing EVs, which are likely to represent the ectosome population. We cannot readily detect the smaller, endosome EVs in our flow cytometry experiments, and draw no conclusions either way regarding any potential role for lipid rafts in their release.

We have used the Ca^2+^ ionophore, A23187, to trigger PS-exposing EV release. This is not a physiological stimulus for platelets. However, we chose A23187 over more physiological activators such as thrombin or collagen for two reasons. First, PS-exposing EVs are released by PS-exposing platelets^7,55^. Not all platelets expose PS when physiological activators are used, even in combination^56,57^. In contrast, A23187 can induce PS exposure in the entire platelet population. Second, using a Ca^2+^ ionophore means that receptor activation and subsequent Ca^2+^ signalling mechanisms are bypassed. Disrupting lipid rafts may affect this signalling if physiological activators were used. Many platelet receptors are found in lipid rafts, including P2Y12 and TPα^36,40,41^. The major collagen receptor, GPVI, is recruited to lipid rafts on stimulation^58^ and lipid rafts regulate platelet activation by GPVI agonists^59^. Important signalling proteins are localised in lipid rafts, such as type I protein kinase PKA^60^, and TRPC6^61^, which is important for agonist-induced PS exposure^57^. Store-operated Ca^2+^ entry, another major pathway for Ca^2+^ entry leading to PS exposure^62^, is also dependent on intact lipid rafts^63^. We did observe that MβCD inhibited release of PS-exposing EVs in response to CRP-XL plus thrombin, which suggests that lipid rafts may also be important in this process in platelets stimulated with physiological activators. However, PS exposure in platelets was also significantly inhibited. The inhibition of release of PS-exposing EVs may reflect inhibition of PS exposure, a process that is required for EV formation. TMEM16F itself does not appear to be affected by cholesterol depletion, since A23187-induced PS exposure was not affected by MβCD. Rather, cholesterol depletion is likely to affect the receptors, ion channels and signalling molecules required for triggering PS exposure in response to physiological activators.

In summary, we have demonstrated that cholesterol-rich lipid rafts are essential for Ca^2+^-triggered release of PS-exposing EVs from platelets. One potential future approach would be to compare the proteomes of platelet lipid rafts and of PS-exposing EVs to identify novel regulators of EV release.

## Methods

### Washed platelet preparation

Blood was drawn by venepuncture from healthy, drug-free volunteers, who had given written, informed consent in accordance with the Declaration of Helsinki. Use of human blood for these experiments was approved by the Human Biology Research Ethics Committee, University of Cambridge. Sodium citrate (3.8% v/v) was used as anticoagulant. Acid citrate dextrose (ACD; 85 mM tri-sodium citrate, 71 mM citric acid, 111 mM D-glucose) was added (1:7 v/v) and platelet-rich plasma (PRP) separated by centrifugation (200 g, 10 min, room temp., no brake). Prostaglandin E_1_ (100 nM) and apyrase (Grade VII; 0.02 U/ml) was added to PRP to prevent platelet activation during preparation. Platelets were pelleted from PRP by centrifugation (600 g, 10 min, room temp., with brake) and resuspended in HEPES-buffered saline (135 mM NaCl, 10 mM HEPES, pH 7.4.) as a density of 5 × 10^7^ platelets/ml. Platelets were rested (30 °C, 30 min) prior to treatment with inhibitors or stimulation. CaCl_2_ (2 mM) was added immediately prior to simulation.

### Flow cytometry

Platelets were treated with inhibitors as indicated in the *Results* section, followed by stimulation with A23187 (10 min). Samples were stained with FITC-conjugated annexin V-FITC (Abcam, Cambridge, U.K.), to detect exposed phosphatidylserine, and PE-Cy7-conjugated anti-CD41 antibody (eBioscience, ThermoFisher, U.K.), to distinguish platelet-derived events. Samples were analysed using a BD Accuri C6 flow cytometer. PE-Cy7 fluorescence (FL3) was used to trigger event acquisition. PS-positive platelet-derived EVs were defined as CD41^+^/annexin V^+^ events that were smaller than 1 µm. The 1 µm gate was set in forward scatter (FSC) using 1 µm silica beads.

### Confocal imaging

Platelets (1 × 10^8^ /ml) treated with vehicle, MβCD or αCD were fixed with 1% paraformaldehyde (PFA; Alfa Aesar), washed by centrifugation (1800 rpm, 10 minutes) resuspended in 250 μl phosphate-buffered saline then adhered to poly-L-Lysine-coated coverslips (0.01 mg/ml; overnight, 4 °C). After gentle washing, coverslip-attached platelets were stained with 50 μg/ml filipin in PBS with 33% (v/v) fetal calf serum (2 hours, room temperature). Coverslips were mounted onto glass plates using Fluoromount-G (0100-01, Southern Biotech). Images were taken using a Leica SP5 confocal microscope, objective 63 × oil, 405 nm excitation and 411–505 nm emission. For quantitative analysis of filipin-stained platelets, images were obtained under identical conditions. Average intensity of fluorescence per platelet was measured using Fiji^64^.

### Western blotting

Platelet lysates that contain 20 μg proteins were electrophoresed on 6% polyacrylamide gels for 45 minutes at 200 V. Proteins were transferred from gels onto 0.45μm polyvinylidene difluoride (PVDF) membranes (IPVH00010, Immobilon-P) for 1 hour at 100 V. Membranes were blocked with 5% w/v non-fat powdered milk in Tris-buffered saline-Tween (TBS-T; 137 mM NaCl, 20 mM Tris, 0.1% Tween-20, pH 7.6) for 1 hour at room temperature, incubated overnight with agitation at 4 °C with 1:10,000 anti-talin antibody (clone 8D4; T3287, Sigma) or 1:5000 anti-CD41 antibody (ab134131, Abcam) and washed three times with TBS-T. To detect the primary antibody, membranes were incubated for 1 hour at room temperature with horseradish peroxidase (HRP)-conjugated anti-rabbit IgG (7074 S, Cell Signaling Technology) or anti-mouse IgG (7076 S, Cell Signaling Technology), again washed three times with TBS-T. To visualise the blot, membranes were exposed to Super Signal Chemiluminescent Substrate (34077, Thermo Scientific) for 5 minutes. The blots were exposed to X-ray films (Amersham Hyperfilm ECL, GE Healthcare) and developed using an OptiMax X-ray film processor (Protec Medizintechnik).

### Transmission electron microscopy

Platelet suspensions were centrifuged to pellet platelets (600 g, 10 minutes). EV-rich supernatants were absorbed onto glow-discharged carbon film-coated 400 mesh copper grids for 3 minutes, washed twice with distilled water, allowed to dry at room temperature, and negatively stained with 3% (w/v) uranyl acetate in water for 30 seconds. Imaging was performed using a Tecnai G2 transmission electron microscope at the Cambridge Advanced Imaging Centre.

### Sources of materials

All reagents were obtained from Sigma (Poole, Dorset, U.K.) unless otherwise stated above. A23187 was from Acros Organics (Fisher Scientific, U.K.). Cangrelor (AR-C 69931MX) was from Selleck Chemicals (Stratech Scientific, Ely, U.K.). Cross-linked collagen-related peptide (CRP-XL) was synthesised by Dr Malcor according to previously published methods^65^.

### Data analysis

Data are reported as mean +/− standard error of mean (SEM) from at least 5 independent platelet preparations, and compared using one-way or two-way analysis of variance (ANOVA), as appropriate, in GraphPad Prism v5. Concentration-response curves were fitted using a four-parameter logistical equation.

### Data availability

The data are available from the corresponding author on reasonable request.

## Electronic supplementary material

Supplementary Fig. 1
