# Supplementary material for: Lipid rafts are essential for release of phosphatidylserine-exposing extracellular vesicles from platelets
Source: Sci Rep. 2018 Jul 3;8:9987. doi: 10.1038/s41598-018-28363-4 (PMC6030044; doi:10.1038/s41598-018-28363-4)
Supplement: Supplementary file 1 — Supplementary Fig. 1 [file 41598_2018_28363_MOESM1_ESM.pdf]

Lipid rafts are essential for release of phosphatidylserine-exposing extracellular vesicles from platelets

Hao Wei, Jean-Daniel M. Malcor, Matthew T. Harper

Supplementary Figure 1

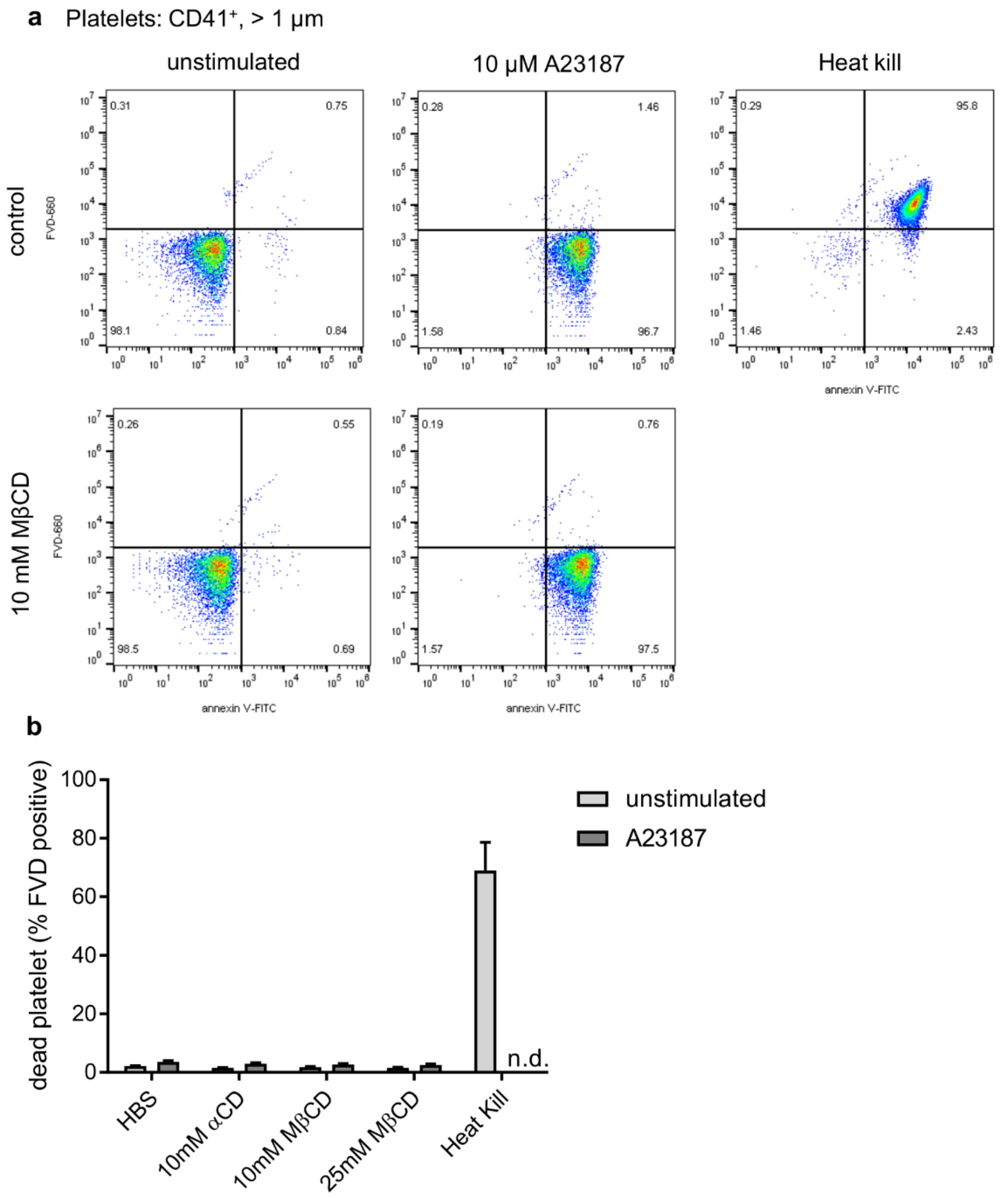

Supplementary Figure 1: MβCD does not affect platelet viability

Platelets were treated with MβCD (10 mM or 25 mM), αCD (10 mM) or vehicle as control, prior to stimulation with 10 µM A23187 for 10 minutes. After this, platelets were stained with anti-CD41-PerCP-Cy7, annexin V-FITC to detect PS exposure, and FVD-660 to detect plasma membrane permeability. Heat-killed platelets were used as a positive control for loss of viability. Representative density plots are show in (a), with FVD-660 fluorescence on the vertical axis, and annexin V-FITC fluorescence on the horizontal axis. Events are CD41+, > 1 µm. (b) Mean ± SEM (n = 5) of % platelets in upper right quadrant (annexin V^+, FVD-660^+). n.d. : not determined.

Supplementary Figure 2

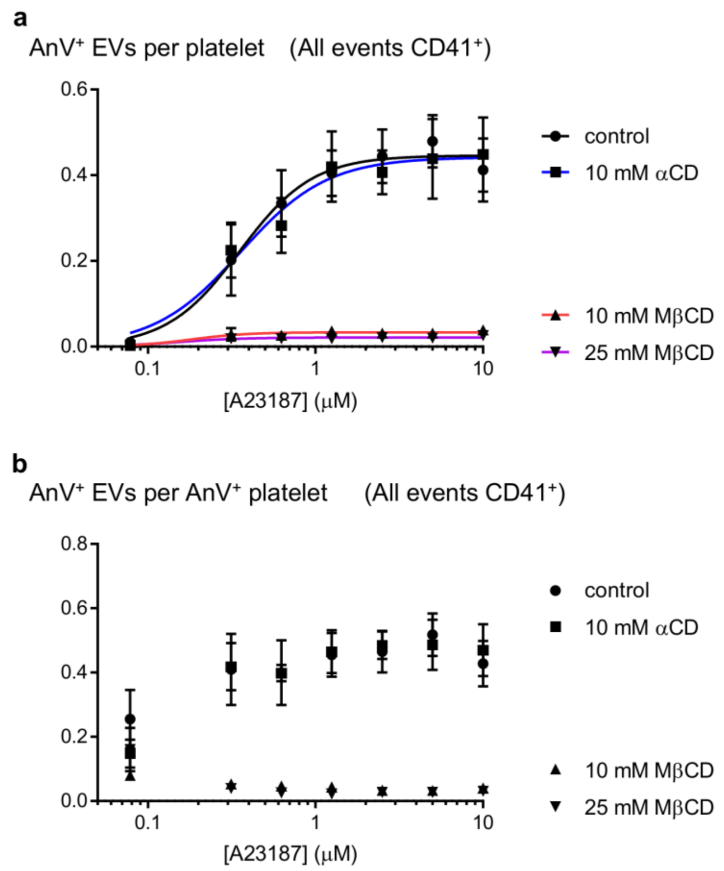

Supplementary Figure 2: Replotting of data in Fig. 2c

To aid interpretation of the density plots in Figure 2, the relationship between AnV+/CD41+ EVs and platelet count is shown. In (a), the data are replotted as number of EVs detected per platelet; in (b), the data are replotted as number of EVs per annexin V+ platelet. Note that at the lower concentration of A23187, there are very few annexin V+ platelets, making it difficult to interpret this ratio in (b). Above this concentration, our flow cytometry assay detects approximately 0.4 AnV+EVs per AnV+ platelet (all events CD41+) in control and αCD-treated samples. In 10 mM MβCD-treated samples, we detect approximately 10-fold fewer (0.04 AnV+ EVs per AnV+ platelet). In samples treated with 25 mM MβCD, we detect only 0.01 AnV^+ EVs per AnV^+ platelet. As discussed in the main text, these values are likely to be underestimates of the total number of EVs released, and probably represent the largest EVs.
